# Supplementary material for: Enhanced emergent electromagnetic inductance in Tb5Sb3 due to highly disordered helimagnetism
Source: Commun Phys. 2024 May 20;7(1):159. doi: 10.1038/s42005-024-01656-z (PMC11106002; doi:10.1038/s42005-024-01656-z)
Supplement: Supplementary file 2 — SUPPLEMENTAL MATERIAL [file 42005_2024_1656_MOESM2_ESM.pdf]

Supplementary Information of

Enhanced emergent electromagnetic inductance in Tb_5Sb_3 due to highly disordered helimagnetism

Aki Kitaori[1,2,3, †], Jonathan S. White[4], Victor Ukleev[4,5], Licong Peng[3],

Kiyomi Nakajima[3], Naoya Kanazawa[6, †], Xiuzhen Yu[3], Yoshichika Ōnuki[3] and

Yoshinori Tokura[2, 3, 7, †]

[1]*Institute of Engineering Innovation, The University of Tokyo, Tokyo 113-0032, Japan*

[2]*Department of Applied Physics, The University of Tokyo, Tokyo 113-8656, Japan*

[3]*RIKEN Center for Emergent Matter Science (CEMS), Wako 351-0198, Japan*

[4]*Laboratory for Neutron Scattering and Imaging (LNS), Paul Scherrer Institute (PSI), CH-5232 Villigen, Switzerland*

[5]*Helmholtz-Zentrum Berlin für Materialien und Energie, D-14109 Berlin, Germany*

[6]*Institute of Industrial Science, The University of Tokyo, Tokyo 153-8505, Japan*

[7]*Tokyo College, The University of Tokyo, Tokyo 113-8656, Japan*

† To whom correspondence should be addressed.

E-mail: kitaori@ap.t.u-tokyo.ac.jp, naoya-k@iis.u-tokyo.ac.jp, and tokura@riken.jp

Supplementary Note 1. Fitting the q-dependence of SANS

To analyze the diffusive streak intensity features of the SANS patterns, we analysed the I (SANS intensity) – q (length of wave vector) curves and found structure in the streak feature that allowed us to identify a magnetic period. The sector box shown in red in Supplementary Fig. 1a ($\theta = 300° \pm 10°$) was used as a representative area of the detector for the analysis. The fitting function applied to the resulting I vs. q curve is $I(q) = A_{FF}\exp\left(\frac{-q^2}{2\sigma_{FF}{}^2}\right) + A_{SM}\exp\left(\frac{-(q-q_0)^2}{2\sigma_{SM}{}^2}\right)$; the first term describes possible ferrimagnetic fluctuations, while the second term corresponds to a magnetic modulation. In these Gaussian fittings, five parameters A_{FF}, σ_{FF}, A_{SM},, Dσ_{SM}, and q_0 were determined based on the Levenberg-Marquardt algorithm. Fig. 1e and Supplementary Fig. 1b-d are comparisons between the actual measurement results and the fitting at T = 2-40 K. In all cases the fitting curves are almost within the range of the error of measurement. Supplementary Fig. 1f shows the temperature dependence of the peak position q_0 determined via this fitting, with the fitted value varying between 0.150 - 0.107 Å^{-1} (λ = 4.19 – 5.88 nm).

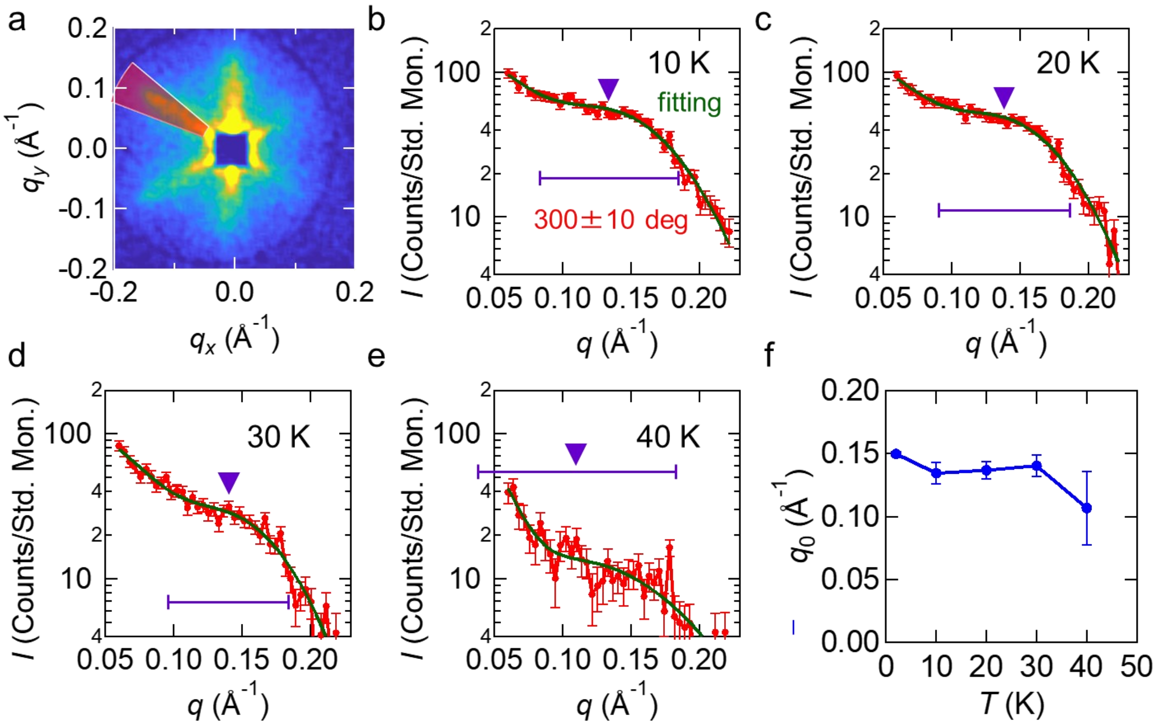

Supplementary Fig. 1 | Fitting the q-dependence of SANS intensity. a, Illustration of the representative analysis sector box on the SANS pattern. The area shaded in red is the $\theta = 300° \pm 10°$ range from the origin ($q_x = q_y = 0$), and the intensity in this region was used for analysis. **b-e**, Dependence of SANS pattern intensity on $q = |\boldsymbol{q}|$ at T = 10 - 40 K. Red symbols are the data, and the dark green curve is the fit. The full width at half-maximum (FWHM) at each point is shown as an error bar. The peak center position and FWHM are indicated with purple triangle and horizontal bar, respectively. **f,** Broad peak position q_0 determined via fitting at each temperature. The magnitude of the error indicates the error range of the peak position determination itself, not the Gaussian standard deviation.

Supplementary Note 2. Lorenz transmission electron microscopy (LTEM) observation of Tb_5Sb_3

Supplementary Figure 2 shows the LTEM images of Tb_5Sb_3 flake at $T = 8$ K. In the thicker (thickness ~ 35 nm) area, there is 1-q stripe pattern. In thinner (thickness ~ 25 nm) area, vortex structures appear to form, possibly due to higher-order exchange interactions characteristic of the thinner film, including dipole-dipole interaction.

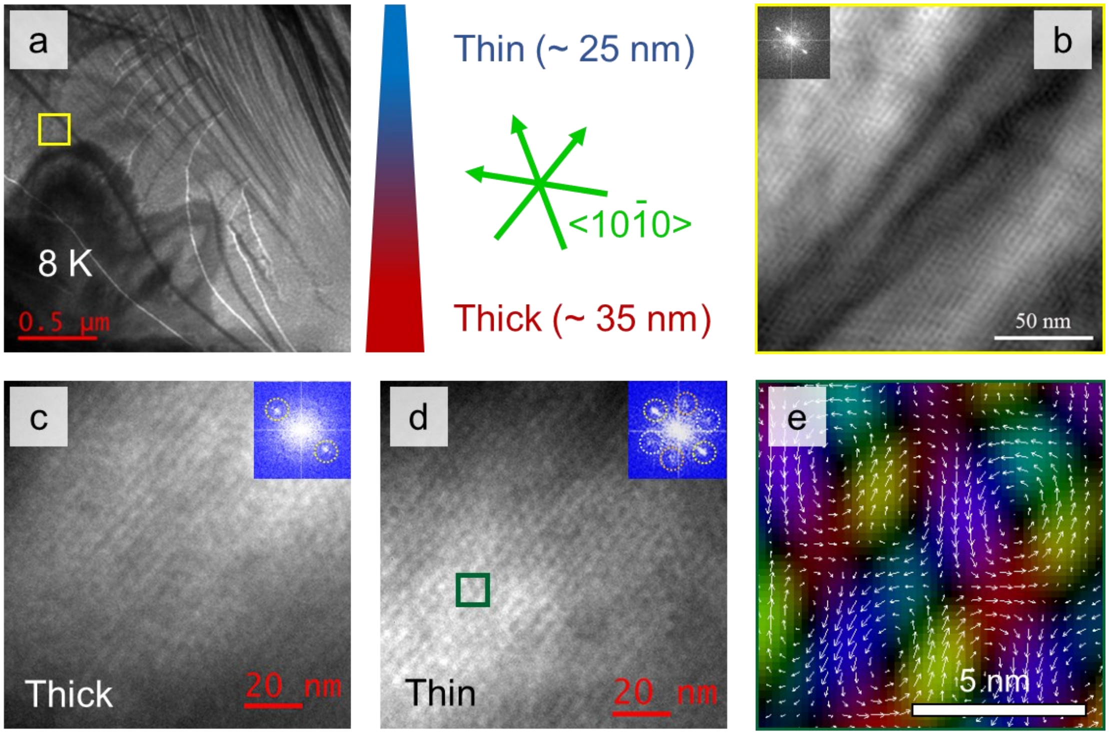

Supplementary Fig. 2 | LTEM images. a, LTEM image observed in the Tb_5Sb_3 flake with a thickness gradient (indicated by a color bar) at $T = 8$ K. The [10-10] direction and its equivalent directions are indicated by green arrows on the right side. **b**, Enlarged view of the yellow framed area in Supplementary Fig. 2a. The top left is a fast Fourier-transformed (FFT) pattern. **c-d**, LTEM images magnified at the thick area (c) and thin area (d), respectively. The upper right insets show the corresponding FFT patterns. They clearly show that the 1-q sate (c) is in the thick region, while the 3-q state (d) is in the thin region. **e**, The magnetic induction map showing magnetization textures for the surrounding area indicated by a green box in Supplementary Fig. 2d.

Supplementary Note 3. Magnetic field dependence of transport

We show the magnetic field ($\mu_0 H$ // c-axis) dependence of transport properties in Supplementary Fig. 3. The top panels, the middle panels, and the bottom panels correspond to real part of the complex resistivity (Re ρ), imaginary part of the complex resistivity (Im ρ), and imaginary part of the 3rd harmonics complex resistivity (-Im ρ^{3f}), respectively. The magnetic transition at $\mu_0 H$ = 4 T observed in SANS appears as a termination of the field hysteresis. Im ρ and -Im ρ^{3f} exhibit qualitatively similar magnetic field dependence, as often observed for the emergent inductance with high nonlinear response[1-3]. The proportion of the nonlinear component in the origin of Im ρ can be evaluated through the ratio with the magnitude of -Im ρ^{3f}. At each temperature, the magnitude of -Im ρ^{3f} is as large as 30 % of that of Im ρ, confirming that the emergent induction is a highly current nonlinear process, as already demonstrated by the current-density dependence of the inductance signals.

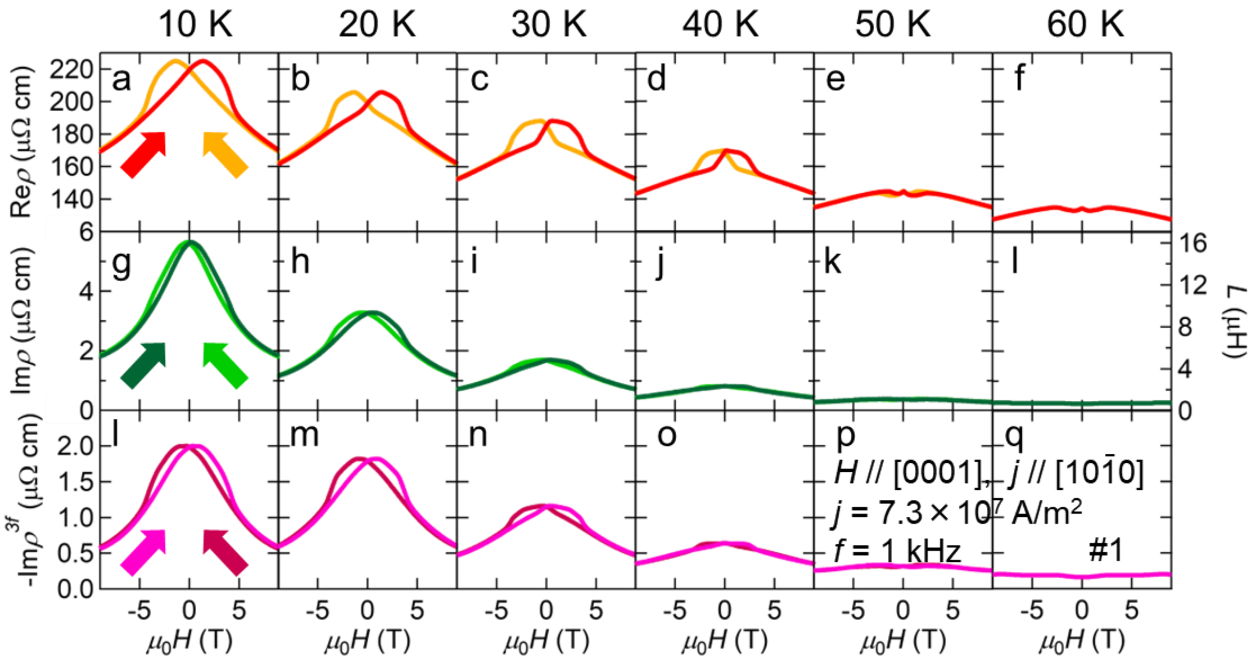

Supplementary Fig. 3 | Magnetic-field dependence of resistivity. a-q, Magnetic-field dependence of the (a-f) real part of the complex resistivity, (g-l) imaginary part of the complex resistivity, (l-q) imaginary part of the 3rd harmonics complex resistivity measured under $H \parallel c$-axis and an ac input current density $j = j_0 \sin(2\pi f t)$ ($j_0 = 7.3 \times 10^7$ A/cm^2, f = 1kHz, $j \parallel a$-axis) at various temperatures.

Supplementary References

1. Yokouchi, T., Kagawa, F., Hirschberger, M., Otani, Y., Nagaosa, N., & Tokura Y. Emergent electromagnetic induction in a helical-spin magnet. *Nature* **586**, 232 (2020).
2. Kitaori, A., Kanazawa, N., Yokouchi, T., Kagawa, F., Nagaosa, N. & Tokura, Y. Emergent electromagnetic induction beyond room temperature. *Proc. Natl. Acad. Sci. USA* **118**, e2105422118 (2021).
3. Kitaori, A., White, J. S., Kanazawa N., Ukleev V., Singh D., Furukawa Y., Arima T., Nagaosa N., & Tokura Y. Doping control of magnetism and emergent electromagnetic induction in high-temperature helimagnets. *Phys. Rev. B* **107**, 024406 (2023).
